# Cross-Study of Malaria Prevalence in History, Bed Net Utilization, and Knowledge about the Disease among Tanzanian College Students

**DOI:** 10.1155/2018/8137051

**Published:** 2018-01-03

**Authors:** Yakobo Nyahoga, Zanda Bochkaeva

**Affiliations:** University of Dodoma, P.O. Box 259, Dodoma, Tanzania

## Abstract

University campuses are potential reservoirs of infectious diseases, but they are not in the research focus. It is obvious that the use of malaria preventive tools is extremely necessary in campus conditions in endemic countries. This study is the first malaria survey, conducted in a student campus in Tanzania. This cross-sectional study uncovered a surprisingly high prevalence of malaria history among students: 89,4% of 246 random respondents assume that they had malaria in history, among whom 145 (58,9%) suffered from the disease during the last year. And although students are relatively confident about the vector, parasite, and prevention measures of the disease, only 44,7% of the students use bed nets and 4,5% use a body spray or ointment daily. The others seldom use spray or ointment or do not care about the problem at all. This situation was found to be associated with two factors, financial and educational. Current results show that students are relatively educated on malaria, but they do not follow the malaria prevention guidance. It has become clear that at least proper informational propaganda of bed net use is required in Tanzanian university campuses.

## 1. Background

In 2016, the World Health Organisation (WHO) reported a significant decrease in the number of malaria cases worldwide. In particular, Tanzania recorded a 75% decline in malaria incidence over the previous 5 years [1]. This decrease is ascribed to the positive outcomes of multiple national and international campaigns, amongst which were “the National Malaria Control Programme” and “Roll Back Malaria” [1–4]. Notwithstanding the above, high levels of personal malaria awareness and individual application of protective measures are critical for reducing infection rates, making continued studies on malaria knowledge, attitudes, and prevention practices very important.

Most malaria investigations in Tanzania have been conducted in rural regions, largely missing out students living on university campuses [5–7]. Nevertheless, university campuses are potential reservoirs of infectious diseases, as is any place where people from different regions live in close proximity for long periods. In the campus environment, the use of insecticide-treated bed nets (ITN) would appear to be the simplest and cheapest method of personal protection from malaria. The efficiency of bed net use is well established, and it is highly recommended for use in endemic regions [8, 9]. Prestudy, informal discussions with students indicated unfortunate and unexpected ignorance of malaria prevention measures and lack of personal application of malaria measures by students at the University of Dodoma (UDOM). This study aimed to investigate the overall perception of ITN use among students of UDOM, as well as identify key factors associated with the ignorance of protection from the disease.

## 2. Materials and Methods

### 2.1. Study Site

The UDOM is the largest university in Tanzania with some 20,000 students from all over the country, studying in seven colleges on a single campus. There is a small health dispensary on campus for students and university staff. Usually, students live on campus, travelling home twice a year for vacations.

The climate of Dodoma region is defined as semiarid with a rainy season lasting from November to April. The peak incidence of malaria cases is during and immediately after the rainy season.

### 2.2. Study Design, Exclusion Criteria, and Data Analysis

A cross-sectional survey was conducted amongst UDOM students between February and March 2017. Some 270 students submitted questionnaires anonymously. 246 questionnaires were analysed. Twenty-four respondents were excluded from the study because of incomplete answers. Thirty-four (34) to forty (40) participants from each college were randomly sampled, based on the distribution of respondents from each college. 56.1% of the respondents were males and 43.9% were females, giving an approximate equivalence of participation based on gender.

The questionnaire was composed of closed and semiclosed questions. Domains across the questionnaire included basic personal information, general well-being, and financial status, as well as malaria literacy and use of prevention measures. Questionnaires were printed on 2 separate sheets of paper and distributed among students to be returned on the next day. Unfortunately, some confusion among students resulted in the personal information being returned separately from the malaria literacy and prevention information, preventing the analysis of the relationship between bed net use and some socioeconomic characteristics.

Frequencies and percentages were summarized and analyzed using Microsoft Excel. Statistical correlations were calculated using the online “Free Statistical Calculator” at https://www.medcalc.org.

### 2.3. Ethical Issues

The study was approved by the University of Dodoma, College of Natural and Mathematical Sciences. Oral informed consent was obtained from all students prior to their involvement in the study and prior to answering the provided questionnaires.

## 3. Results

### 3.1. Socioeconomic Characteristics of the Group

**Figure 1:**
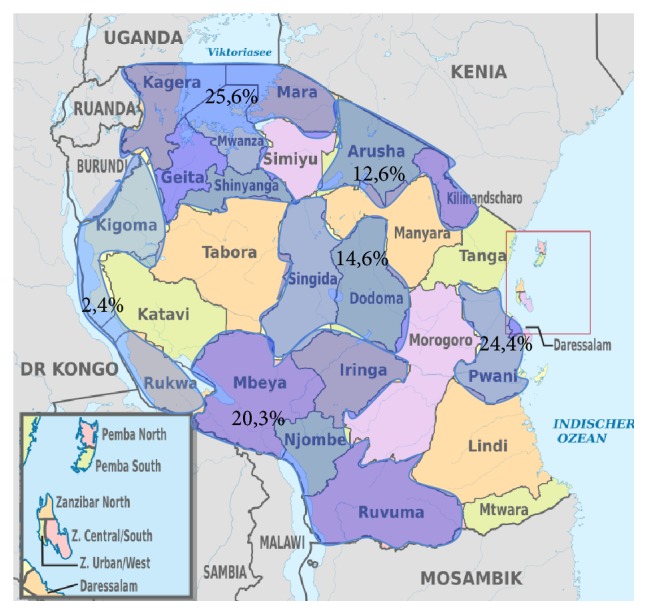
Percentage distribution of regions where respondents are originally from.

Among 246 recruited students, 138 (56.1%) were females and 108 (43.9%) were males. The majority of respondents were 20–25 years of age, single, and with no children. Almost all of them did not smoke (97%) and did not drink alcohol regularly (89.1%), and most of them (66.7%) exercised at least once a week. Interrogated respondents were originally from the lake zone (Mwanza, Mara, Kagera, and Shinyanga), the northern zone (Arusha and Kilimanjaro), the southern and southern highlands zone (Mbeya, Iringa, Njombe, and Ruvuma), the eastern zone (Dar es salaam and Pwani), the central zone (Dodoma and Singida), and the western zone (Rukwa and Kigoma) (Figure 1). More than half of the respondents (55%) had a monthly income of less than 200,000 Tshs (about 90$), 69 students (28%) had from 200,000 to 500,000 Tshs (90–220$), and the income of the remaining 17% was more than 220$ (see Table 1).

### 3.2. Prevalence of Malaria in History and Knowledge about the Disease

The prevalence of malaria infection in individual histories showed striking results: 220 (89.4%) students answered positively, an incredibly large proportion. Within this group, 145 (59%) reported suffering from infection within the last year.

The knowledge about malaria is relatively high: 98% of the students demonstrated confidence about the vector and 87.8% about preventive measures. Regarding knowledge of the causative agent, only 65.8% identified* Plasmodium* as an intracellular parasite, with the remainder believing that it is a bacterium (24.6%) or virus (8.8%), and one student answered that malaria is a genetic disorder.

### 3.3. Use of Mosquito Bed Nets

110 (44.7%) students reported that they sleep under a bed net on campus, 33 (13.4%) use antimosquito sprays or ointments regularly, and the remainder do not protect themselves from malaria infection. At the same time, 188 (76.4%) agreed that sleeping under an ITN is the most effective approach for disease prevention, 11 (4.5%) students thought sprays and ointments are the most effective, and the remainder considered that only antimalarials could help (20 or 8.1%) or did not answer this question (27 or 11%). 107 out of 136 (78.7%) non-ITN-using students have a monthly income of not more than 90$, and 87 (64%) of this group considered that they are not at risk of infection with the* Plasmodium* parasite in Dodoma region.

## 4. Discussion

### 4.1. Socioeconomic Description of the Study Group and Common Knowledge about Malaria

Socioeconomic characteristics of the study group can be described as usual for Tanzanian students, showing a surprisingly high percentage of students who take care of their health: almost none of them smoke or drink alcohol and two-thirds do weekly exercise. These statistics correlate with the data presented by Amemori, where drinking and smoking habits among university students were investigated [10].

The sample group included respondents from the majority of Tanzanian regions, allowing us to draw a reasonably accurate picture of educated youth throughout the country. Additionally, World Bank data indicates that only 5% of Tanzanians have a university education and the sample group belongs to this small community of Tanzania's most educated citizens [11]. Potentially, today's students are tomorrow's leaders of Tanzania. Considering that the focus of the study was Tanzania's young, educated elite, levels of literacy about the malaria vector and preventive measures were as high as expected and only knowledge about the biology of the* Plasmodium* parasite could have been higher, especially for an endemic country—29.2% of the respondents do not know that it is an intracellular parasite. This would probably be insignificant had it not been for the frequency with which preventative measures are neglected by individuals. Perhaps a greater depth of knowledge of the biology of the parasite and the severity and prevalence of the disease would result in greater individual application and care with preventative measures.

### 4.2. Malaria in History

The high prevalence of malaria in history may not accurately reflect reality. Even recently and certainly historically, the lack of proper diagnostic facilities, as well as the absence of medical insurance, led to any fever, fatigue, or other symptoms being diagnosed as malaria [12]. Additionally, it is possible that the observed prevalence of malaria in history arises from self-diagnosis and self-treatment in childhood. Nowadays, diagnostics have become available in any dispensary, and the cost of diagnostics (discussed further below) is much lower than treatment, so people prefer to make sure about the* Plasmodium* infection before buying expensive antimalarials. From this point of view, it is important to note that at least 145 students (58.9%) suffered from the disease during the last year and were potentially infectious while living on campus. It follows that the campus can be considered as a high-risk area and the use of bed nets is critically necessary.

### 4.3. ITN Use and Factors Associated with the Ignorance of Malaria Prevention

The percentage of ITN use among students is 44.7%, which is lower than in rural regions of Tanzania [13–15]. The primary factor of ignorance of preventive measures is predictable. Malaria is a poverty-associated disease, so most of the students who are not using bed nets are in the lowest income group of the sample (OR = 19.03, 95% CI: 9.55–38) (see Table 2). Moreover, students have never experienced the LLIN distribution campaigns, and they live in Dodoma region (on campus) which they erroneously think to be of low risk, so they feel that they are safe and want to save money on prevention tools. It should be noted that a bed net costs about 5$ in Tanzania, and if used properly, even without retreatment, it can protect from mosquito bites effectively enough for years. In comparison, the total cost of a diagnostic test and antimalarials is higher, at 2$ and 7$, respectively. Plainly, the cost of one confirmed bout of malaria in one year is twice as much as the cost of one bed net potentially protecting from infection for several years.

The second factor, found to be associated with the ignorance of malaria protection, is the opinion that they are not at risk of infection (OR = 22.64, 95% CI: 10.17–50.4). Evidently, there is a strong correlation between the use of bed nets and the awareness about the risk of malaria infection. Unfortunately, the prevalence of bed net use is far from 100%, not only among students, but also in the whole country. Although the prevalence of malaria in Dodoma region is as low (reported in 2015—about 3%), the continued use of bed nets is required to eliminate the infection or keep it down [16].

Interestingly, no correlation between historical rates of bed net use and malaria was found, probably due to lack of data, and because the timeline of bed net use and malaria rates is unknown. This correlation needs to be investigated.

## 5. Conclusion

The recent significant success in the fight against malaria could give the false impression that the disease has been almost overcome. The reported decrease in mortality and case incidence only reflects the efficiency of the reported strategies. Much remains to be done. These data demonstrate terrifyingly low levels of prevention practice (bed net use) together with high levels of historical infection among students, a group that is usually left outside the focus of research. Students are relatively well informed about malaria, but even so, they do not follow malaria prevention guidance. Perhaps students' knowledge about malaria is memorized by them in an educational sense, but they do not transfer and apply this knowledge on a practical or personal level for individual disease prevention. It is clear that at least proper informational propaganda of ITN use is required in university campuses and that the student community should be continuously monitored to fill in gaps in the data and clarify malaria infection rates in what may well be a local, high-risk environment.

## Figures and Tables

**Table 1:** Sociodemographic characteristics of the study group.

	Number (total: 246)	%
Gender		
Male	138	56,1
Female	108	43,9
Age group (years)		
Under 20	15	6,1
20–25	188	76,4
26–30	32	13
Above 30	11	4,5
Marital status		
Single	182	74
Married	17	6,9
Having a regular partner	47	19,1
Children		
0	221	89,8
1 or 2	23	9,3
3 to 5	2	0,8
Exercise		
0	82	33,3
1 to 2	110	44,7
3 to 5	37	15
6 to 7	17	6,9
Smoking regularly		
Yes	8	3,3
No	238	96,7
Drinking alcohol at least once a week		
Yes	27	11
No	219	89
Monthly income in USD		
<90	123	50
90–220	69	28
220–320	20	8,1
320–450	11	4,5
Refused to answer	23	9,3
Risk of having malaria		
Yes	151	61,4
No	95	38,6
History of malaria		
Yes	220	89,4
During the last year	145	58,9
No	20	8,1
Do not know	6	2,4
Bed net use		
Yes	110	44,7
No	136	55,3
Spray or ointment	11	4,5
Malaria causative agent		
Bacterium	52	21,1
Intracellular parasite	174	70,7
Virus	19	7,7
Your variant	1	0,4
Effective preventive measures		
Bed net	188	76,4
Spray or ointment	11	4,5
Antimalarials	20	8,1
No answer	27	11

**Table 2:** Correlation between ITN use and monthly income and awareness about malaria risk.

	Total	Sleep under the bed net		OR	95% CI
		Yes	No		
Monthly income in USD					
<90	123	16	107	19.03	9.55 to 38
≥90	100	74	26		
Risk of having malaria					
Yes	151	102	49	22.64	10.17 to 50.4
No	95	8	87		
